# Recurrent Thrombotic Events after Discontinuation of Vitamin K Antagonist Treatment for Splanchnic Vein Thrombosis: A Multicenter Retrospective Cohort Study

**DOI:** 10.1155/2015/620217

**Published:** 2015-10-05

**Authors:** Nicoletta Riva, Walter Ageno, Daniela Poli, Sophie Testa, Serena Rupoli, Rita Santoro, Teresa Lerede, Antonietta Piana, Monica Carpenedo, Alberto Nicolini, Piera Maria Ferrini, Giuliana Martini, Catello Mangione, Laura Contino, Carlo Bonfanti, Paolo Gresele, Alberto Tosetto

**Affiliations:** ^1^Department of Clinical and Experimental Medicine, University of Insubria, 21100 Varese, Italy; ^2^Department of Heart and Vessels, Thrombosis Centre, AOU Careggi, 50134 Florence, Italy; ^3^Haemostasis and Thrombosis Centre, AO Istituti Ospitalieri, 26100 Cremona, Italy; ^4^Division of Hematology, Haemostasis and Thrombosis Center, AOU Ospedali Riuniti Umberto I, G.M. Lancisi, G. Salesi, 60020 Ancona, Italy; ^5^Hemophilia Center, Hemostasis and Thrombosis Unit, Azienda Ospedaliera “Pugliese-Ciaccio”, 88100 Catanzaro, Italy; ^6^Department of Immunohematology and Transfusion Medicine, Hospital Papa Giovanni XXIII, 24100 Bergamo, Italy; ^7^Department of Internal Medicine, Ospedale San Martino, 16100 Genoa, Italy; ^8^Department of Hematology and Transplant Unit, AO San Gerardo, 20900 Monza, Italy; ^9^Department of Internal Medicine, Arcispedale Santa Maria Nuova, 42100 Reggio Emilia, Italy; ^10^Dipartimento Emergenza-Urgenza e Area Medica e Specialistica, AOU Parma, 43100 Parma, Italy; ^11^Spedali Civili di Brescia, 25100 Brescia, Italy; ^12^Ospedale di Galatina, 73013 Galatina, Italy; ^13^Dipartimento Onco-Ematologico e Medicina Specialistica, Azienda Ospedaliera Nazionale SS. Antonio e Biagio e Cesare Arrigo, 15100 Alessandria, Italy; ^14^Department of Transfusion Medicine and Haematology, Carlo Poma Hospital, 46100 Mantua, Italy; ^15^Department of Internal Medicine, University of Perugia, 06126 Perugia, Italy; ^16^Department of Hematology, Hemostasis and Thrombosis Center, S. Bortolo Hospital, 36100 Vicenza, Italy

## Abstract

It is generally recommended that patients with splanchnic vein thrombosis (SVT) should receive a minimum of 3 months of anticoagulant treatment. However, little information is available on the long-term risk of recurrent thrombotic events. The aim of this study was to evaluate the risk of venous and arterial thrombosis after discontinuation of vitamin K antagonist (VKA) in SVT patients. Retrospective information from a cohort of SVT patients treated with VKA and followed by 37 Italian Anticoagulation Clinics, up to June 2013, was collected. Only patients who discontinued VKA and did not receive any other anticoagulant drug were enrolled in this study. Thrombotic events during follow-up were centrally adjudicated. Ninety patients were included: 33 unprovoked SVT, 27 SVT secondary to transient risk factors, and 30 with permanent risk factors. During a median follow-up of 1.6 years, 6 venous and 1 arterial thrombosis were documented, for an incidence of 3.3/100 patient-years (pt-y). The recurrence rate was highest in the first year after VKA discontinuation (8.2/100'pt-y) and in patients with permanent risk factors (10.2/100'pt-y). Liver cirrhosis significantly increased the risk of recurrence. In conclusion, the rate of recurrent vascular complications after SVT is not negligible, at least in some patient subgroups.

## 1. Introduction

Splanchnic vein thrombosis (SVT) involves one or more abdominal veins (portal, mesenteric, splenic, and suprahepatic veins) [1] and carries a substantial risk of bowel ischemia and chronic portal hypertension. The risk of recurrent venous thromboembolism (VTE) has been reported to range from 2.3 to 3.5 per 100 patient-years [2, 3], with approximately half of these events involving the splanchnic veins and the other half involving the limb veins or the pulmonary arteries. Furthermore, myocardial infarction and ischemic stroke are also common in SVT patients and the cumulative incidence of arterial and venous thrombotic events after SVT has been estimated to be 5.5 per 100 patient-years [4]. However, these data included both on-treatment patients and off-treatment patients.

The optimal duration of secondary prevention of SVT is still a matter of debate. In previous studies, prothrombotic states (such as thrombophilic abnormalities, myeloproliferative neoplasms, or hormonal therapy) emerged as risk factors for recurrence, while there was no agreement on the role of the anticoagulant treatment [5]. In the presence of a reversible provoking risk factor (e.g., recent surgery, oral contraceptive, and abdominal sepsis), current guidelines suggest discontinuing anticoagulant treatment after 3 months, as for usual site VTE [6]. Vice versa, in the presence of a persistent risk factor (e.g., myeloproliferative neoplasms) or unprovoked SVT, or in patients with particularly severe thrombosis (e.g., Budd-Chiari syndrome), extended anticoagulation is suggested, with periodic reassessment of the bleeding risk [6, 7].

The aim of this study was to evaluate the risk of recurrent thrombotic events after discontinuation of vitamin K antagonist (VKA) treatment in patients with splanchnic vein thrombosis.

## 2. Materials and Methods

### 2.1. Study Population and Outcomes

This study is part of a project [8] involving 37 centers affiliated to the Italian Federation of Anticoagulation Clinics (see Appendix for the full list of participating FCSA centers and collaborators). The FCSA centers were requested to retrospectively review the charts of all adult patients with objectively diagnosed SVT (portal, mesenteric, splenic, or suprahepatic) that were treated with VKA and attended these anticoagulation clinics until June 2013. For the purpose of the present substudy, we included only patients who discontinued VKA treatment before the end of the main study. Exclusion criteria were major bleeding events during VKA and prescription of any other anticoagulant drug (such as low molecular weight heparin) after VKA discontinuation. Patients who received antiplatelet drugs were eligible.

In this open-cohort study, follow-up was started at the time of VKA interruption (which occurred between 2000 and May 2013) and it was stopped after the first vascular event or after death occurred, at the end of the study (June 2013), or when a patient was restarted on anticoagulant treatment for any other reason.

Baseline patients' characteristics (demographics and risk factors) were collected at the time of SVT diagnosis, together with information on clinical onset and on the involved veins. SVT was classified as secondary to transient risk factors (recent surgery, intra-abdominal infection, use of hormone therapy, and abdominal trauma), to permanent risk factors (solid cancer, myeloproliferative neoplasm, liver cirrhosis, and inflammatory bowel disease) or as unprovoked. Patients with both a transient and a permanent risk factor were considered in the second category. Study data were collected and managed using REDCap electronic data capture software [9].

All vascular complications during follow-up were centrally reviewed by an independent adjudication committee and classified as venous thrombotic events (SVT recurrence in a recanalized or previously not involved vein, deep vein thrombosis (DVT), and pulmonary embolism (PE)) or arterial thrombotic events (acute coronary syndrome, stroke, and transient ischemic attack).

This study is reported following the Strengthening the Reporting of Observational Studies in Epidemiology (STROBE) statement for observational studies [10].

### 2.2. Statistical Analysis

Continuous variables were reported as median with interquartile range (IQR), categorical variables as counts and percentages. Follow-up time was considered “off-treatment,” computed from the discontinuation of VKA until the first vascular event or death, study end (June 2013), or when a patient restarted the anticoagulant treatment for any other reason.

The number of vascular complications was expressed as cumulative incidence and as incidence rate, calculated as the number of events per 100 patient-years of observation with 95% confidence intervals (CI). Kaplan-Meier curves were used to estimate the cumulative incidence of vascular complications after VKA discontinuation.

To identify predictors of vascular complications, we performed a multivariable Cox regression analysis using backward stepwise elimination (with levels of *P* < 0.05 for inclusion and *P* > 0.1 for exclusion). We started with the following variables: age, male sex, personal history of VTE, family history of VTE, unprovoked genesis, SVT secondary to transient risk factors, malignancy (solid cancer or MPN), liver cirrhosis, duration of previous VKA treatment, and use of antiplatelet therapy during follow-up. Variables with a *P* value <0.05 at multivariable analysis were considered statistically significant.

The potential effect of antiplatelet drugs on the rate of recurrent thrombotic events was specifically analyzed with an additional univariable Cox regression analysis.

All analyses have been performed using STATA SE 12 (StataCorp LP, College Station, TX, USA).

## 3. Results

### 3.1. Population Characteristics

**Figure 1:**
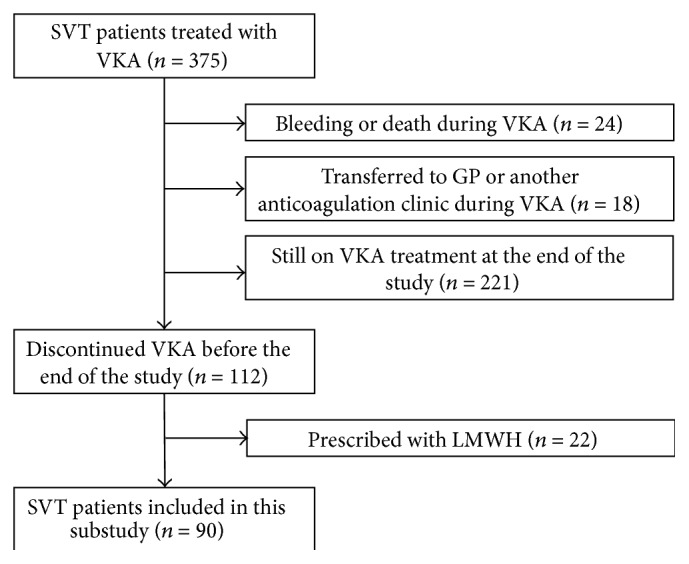
Flow diagram of patients included in this substudy. GP = general practitioner, LMWH = low molecular weight heparin, SVT = splanchnic vein thrombosis, and VKA = vitamin K antagonist.

Overall, 112 (29.9%) of 375 SVT patients treated with VKA discontinued the oral anticoagulant treatment. Twenty-two patients were excluded from this study because they subsequently received low molecular weight heparin, thus leaving 90 patients for this analysis. The flow diagram of patients included in the present study and reasons for exclusion are reported in Figure 1.

Median age was 50 years (IQR 39–62); 52.2% of patients were males. Personal history of VTE was reported in 7.8%, while family history of VTE was reported in 8.9%. In 27 patients SVT was secondary to transient risk factors, in 30 patients it was secondary to permanent risk factors, and in 33 patients SVT was classified as unprovoked. Details regarding the most common risk factors, sites of thrombosis, and other baseline characteristics are reported in Table 1.

All patients had previously received anticoagulant treatment with warfarin for a median duration of 12.4 months (IQR 7.1–23.8) and with a median time within therapeutic range of 62% (IQR 56–75.5). The prescribed INR range was 2.0–3.0 in 93.3% of patients, while a lower range was prescribed in 5.6% and higher in 1.1%.

### 3.2. Follow-Up and Thrombotic Events

The total follow-up off-treatment was 211.8 patient-years and median 1.6 (IQR 0.5–3.3) years. Fourteen patients (15.6%) received antiplatelet therapy for some time during follow-up.

After the central adjudication process, 6 venous thrombotic events (3 SVT recurrences, 2 DVTs of the lower limbs, and 1 renal vein thrombosis) and 1 arterial thrombotic event (acute coronary syndrome) were documented. Details regarding thrombotic events are reported in Table 2. Characteristics of patients with vascular events during follow-up are summarized in Table 3.

**Figure 2:**
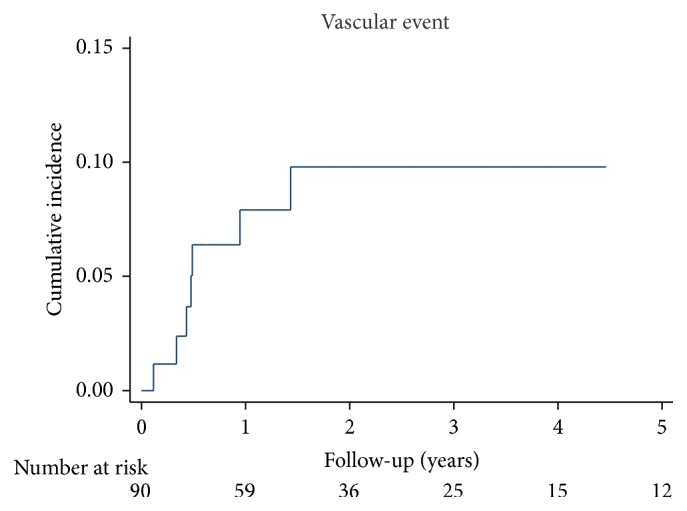
Cumulative incidence of vascular events after discontinuation of vitamin K antagonist treatment.

The cumulative incidence of thrombotic events was 7.8% and the overall incidence rate was 3.3 (95% CI 1.6–6.9) per 100 patient-years. The incidence of thrombotic events was 8.2 (95% CI 3.7–18.3) per 100 patient-years during the first year after VKA discontinuation and 0.7 (95% CI 0.1–5.1) per 100 patient-years thereafter (Figure 2).

**Figure 3:**
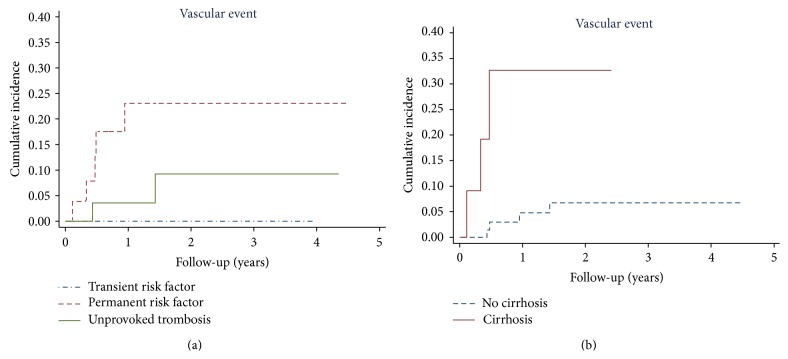
Cumulative incidence of vascular events according to the pathogenesis of splanchnic vein thrombosis (a) and the presence of liver cirrhosis (b).

Of note, the recurrence rate was 2.4 (95% CI 0.6–9.6) per 100 patient-years in patients with unprovoked SVT and 10.2 (95% CI 4.2–24.4) per 100 patient-years in patients with a permanent risk factor, while no thrombotic event occurred in patients with a transient risk factor (Figure 3(a)).

At multivariable analysis, liver cirrhosis emerged as independent predictor of thrombotic complications (hazard ratio (HR) 7.9; 95% CI, 1.8–35.9; *P* = 0.007). The incidence of recurrent vascular events was 19.1 (95% CI 6.2–59.1) per 100 patient-years in patients with liver cirrhosis and 2.0 (95% CI 0.8–5.4) per 100 patient-years in patients without liver cirrhosis (Figure 3(b)).

The use of antiplatelet drugs after VKA discontinuation did not affect the rate of recurrent vascular complications (HR 2.2; 95% CI, 0.4–11.3; *P* = 0.351).

Four patients died during follow-up, corresponding to an incidence rate of 1.9 (95% CI 0.7–5.0) per 100 patient-years. None of these deaths was attributed to thrombotic events and the causes of death were malignancy (*n* = 2), sepsis (*n* = 1), and liver cirrhosis (*n* = 1).

All patients concluded the observational period as planned, without any loss to follow-up. Two patients were restarted on VKA for reasons other than a recurrent event, and they were accounted only for their time off-treatment.

## 4. Discussion

In this study, we specifically aimed at estimating the risk of recurrent thrombotic events after discontinuation of VKA treatment in patients with SVT. During a median follow-up of 1.6 years, we found that the overall incidence of vascular events, including both venous and arterial thrombosis, was 3.3 (95% CI 1.6–6.9) per 100 patient-years. The majority of thrombotic events occurred shortly after VKA discontinuation, with a median time from VKA suspension to recurrence of 0.5 years, which corresponds to an incidence rate of 8.2 (95% CI 3.7–18.3) per 100 patient-years in the first year.

Our study specifically included SVT with different etiology, therefore providing an estimate of the risk of recurrent thrombotic events in the overall population of patients who discontinued VKA for reasons other than bleeding complications. Two previous retrospective studies evaluated the risk of recurrent arterial and venous thrombotic events, but they included only patients with nonmalignant noncirrhotic portal vein thrombosis (PVT). Spaander et al. reported rates of recurrent thrombotic event of 3% at 1 year, 8% at 5 years, and 24% at 10 years, with a median time from PVT diagnosis to recurrence of 5.7 years [11]. Although the anticoagulant treatment was associated with a trend towards a reduction of recurrent thrombosis (HR 0.2 and *P* = 0.1), separate incidence rates off- and on-treatment were not provided [11]. Condat et al. reported an overall incidence rate of 5.5 per 100 patient-years with a constant trend (3.4 per 100 patient-years during the first year, 6.4 during the second year, and 7.7 during the third year) [4]. However, in this study separate rates in patients with and without anticoagulant treatment were reported only for the outcome recurrent PVT (0.64 versus 1.87 per 100 patient-years, resp.) [4].

In the first part of this project, we described 375 SVT patients treated with VKA and, during a median follow-up of 1.98 years, we reported an incidence rate of vascular events on-treatment of 1.37 (95% CI, 0.84–2.23) per 100 patient-years, including 9 venous and 7 arterial thrombotic events [8], while the incidence rate off-treatment from the present study was approximately 2.5-fold higher (3.3 per 100 patient-years). Thus, in our cohort, recurrence rates were similar to those reported in previous studies conducted in more selected populations, although the majority of recurrent thrombotic events occurred shortly after VKA discontinuation, suggesting that SVT patients who discontinue VKA might benefit from a close follow-up.

Another interesting finding in our study was the previous duration of VKA treatment. Our cohort consisted of a selected population of SVT patients, who were prescribed with VKA for a definite period of time. Therefore, patients who were required to discontinue oral anticoagulation because of major bleeding were excluded, as well as patients prescribed with long-term VKA. In contrast to usual site VTE, where approximately 30% of patients were treated for 3 months or less and approximately 50% between 3 and 6 months [12], half of the SVT patients included in our cohort were treated for at least one year. Although current guidelines recommend definite treatment duration in some SVT patients, especially those with transient risk factors, in real life clinical practice unusual site VTE is usually treated for a longer period of time.

The identification of which SVT patients might safely discontinue VKA is of utmost importance. Although our sample size and the number of events were small, we found that patients with transient risk factors did not develop any thrombotic event during follow-up, while the incidence rate was moderate for unprovoked SVT (2.4 per 100 patient-years and 95% CI 0.6–9.6) and high for patients with persistent risk factors (10.2 per 100 patient-years and 95% CI 4.2–24.4). In particular, liver cirrhosis was associated with approximately an 8-fold increased risk of developing thrombotic events. Patients with liver cirrhosis have been excluded from the majority of previous studies evaluating thrombosis in the splanchnic venous system [4, 11], but they have been recently enrolled in specific trials. Delgado et al, in a cohort of 55 cirrhotic patients with PVT, reported high rate of recurrent SVT even in patients who obtained complete recanalization (38.5% with a median time to recurrence of 1.3 months after discontinuation of the anticoagulant treatment) [13]. The authors suggested that anticoagulant treatment should be maintained life-long in cirrhotic patients, due to the persistent underlying pathophysiological mechanism [13]. Villa et al. conducted a randomized controlled trial in patients with advanced liver cirrhosis and compared primary prevention with low molecular weight heparin (enoxaparin 4000 U daily) versus no treatment [14]. They found that enoxaparin was effective in preventing the development of PVT and the occurrence of hepatic decompensation, without a significant increase in the risk of bleeding complications, and suggested that PVT prevention might be less harmful than SVT treatment in cirrhotic patients [14].

Our study has potential limitations that need to be acknowledged. First, we included a selected population of SVT patients discontinuing VKA, after being followed by several anticoagulation clinics. Therefore, a selection bias is possible and our findings might not be generalizable to the entire population of SVT patients or to never-anticoagulated SVT patients. Furthermore, in this retrospective cohort study reflecting SVT management in real life clinical practice, the decision whether to stop the anticoagulant treatment was taken by the attending physicians. As a consequence, a confounding by indication bias is also possible, because patients more likely to develop recurrent thrombosis did not discontinue VKA and therefore were not included. Patients who suspended VKA because of a bleeding complication were also excluded from this study, because of their potential increased risk of vascular events.

Second, the small sample size of our cohort resulted in wide confidence interval and possibly in the lack of statistically significant associations of other risk factors previously reported to be related with SVT recurrence (such as hormonal treatment or myeloproliferative neoplasm). Third, due to the retrospective design of our study, other potential thrombotic risk factors (such as JAK2 mutation) were tested in only a minority of patients. However, the participating centers affiliated to the Italian Federation of Anticoagulation Clinics have a high standard of care and routinely collect clinical information on their patients. Therefore, apart from genetic mutation, no other information regarding risk factor was missing.

The main strengths of our study were the multicenter design, involving several Italian Anticoagulation Clinics, and the special focus on the off-treatment period in a homogeneously untreated cohort of SVT patients after discontinuation of VKA.

## 5. Conclusion

The rate of recurrent vascular events after SVT, including arterial and venous thrombotic events, is not negligible. The incidence rate was particularly remarkable in the first year after VKA discontinuation and in patients with permanent risk factors. Liver cirrhosis was significantly associated with an 8-fold increased risk of recurrence. Our findings need to be confirmed in larger prospective studies.

## Figures and Tables

**Table 1:** Baseline characteristics of the population.

	Patients with SVT (*n* = 90)
Age (years), median (IQR)	50 (39–62)
Males, *n*/*N* (%)	47/90 (52.2%)
Personal history of VTE, *n*/*N* (%)	7/90 (7.8%)
Family history of VTE, *n*/*N* (%)	8/90 (8.9%)
Asymptomatic incidentally detected SVT, *n*/*N* (%)	11/90 (12.2%)
Risk factors^*^	
Unprovoked SVT, *n*/*N* (%)	33/90 (36.7%)
Liver cirrhosis, *n*/*N* (%)	14/90 (15.6%)
Solid cancer, *n*/*N* (%)	6/90 (6.7%)
Myeloproliferative neoplasm, *n*/*N* (%)	7/90 (7.8%)
Recent abdominal surgery, *n*/*N* (%)	10/90 (11.1%)
Hormonal therapy, *n*/*N* female (%)	11/43 (25.6%)
Inflammatory bowel disease, *n*/*N* (%)	5/90 (5.6%)
Other intra-abdominal inflammation or infection, *n*/*N* (%)	7/90 (7.8%)
Involved veins	
Multiple veins thrombosis, *n*/*N* (%)	33/90 (36.7%)^**^
Isolated portal vein thrombosis, *n*/*N* (%)	35/90 (38.9%)
Isolated mesenteric veins thrombosis, *n*/*N* (%)	16/90 (17.8%)
Isolated suprahepatic vein thrombosis, *n*/*N* (%)	1/90 (1.1%)
Isolated splenic vein thrombosis, *n*/*N* (%)	5/90 (5.6%)
Genetic mutations	
JAK2 V617F mutation, *n*/*N* tested (%)	3/42 (7.1%)
Prothrombin G20210A mutation, *n*/*N* tested (%)	4/55 (7.3%)
Factor V Leiden mutation, *n*/*N* tested (%)	5/58 (8.6%)
Previous anticoagulant treatment	
VKA treatment duration (months), median (IQR)	12.4 (7.1–23.8)
Less than 3 months, *n*/*N* (%)	7/90 (7.8%)
3–6 months, *n*/*N* (%)	10/90 (11.1%)
6–12 months, *n*/*N* (%)	22/90 (24.4%)
1 year or more, *n*/*N* (%)	51/90 (56.7%)
Time within therapeutic range (%), median (IQR)	62 (56–75.5)

^*^Multiple risk factors are possible.

^**^Of these 33 patients, 20 had two veins involved (16 portomesenteric venous thrombosis) and 13 had three veins involved (all portosplenomesenteric venous thrombosis).

IQR = interquartile range, SVT = splanchnic vein thrombosis, VKA = vitamin K antagonist, and VTE = venous thromboembolism.

**Table 2:** Vascular events after discontinuation of vitamin K antagonist treatment.

Total follow-up off-treatment	211.8 patient-years
Follow-up time, median (IQR)	1.6 (0.5–3.3) years
Cumulative incidence of vascular events, *n*/*N* (%)	7/90 (7.8%)
Incidence rate of vascular events (95% CI)	3.3 (1.6–6.9) per 100 patient-years
Time elapsed from discontinuation of VKA treatment to the first vascular event, median (IQR)	0.5 (0.3–0.9) years
Type of vascular events	
Venous thrombotic events, *n*	**6**
SVT recurrence, *n*	3^*^
Lower limb proximal or distal DVT, *n*	2
Renal vein thrombosis, *n*	1
Arterial thrombotic events, *n*	**1**
Acute coronary syndrome, *n*	1

^*^Two recurrent SVT were diagnosed incidentally in cirrhotic patients undergoing abdominal imaging as follow-up of their liver disease (portal vein and portomesenteric veins thrombosis); a suprahepatic vein thrombosis was diagnosed in a patient with polycythemia vera presenting with abdominal pain and ascites.

DVT = deep vein thrombosis, IQR = interquartile range, PE = pulmonary embolism, SVT = splanchnic vein thrombosis, and VKA = vitamin K antagonist.

**Table 3:** Characteristics of patients with vascular events during follow-up.

Variable	Patients with vascular events (*n* = 7)	Patients without vascular events (*n* = 83)
Age (years), median (IQR)	47 (31–62)	50 (40–62)
Males, *n* (%)	4 (57.1%)	43 (51.8%)
Personal history VTE, *n* (%)	1 (14.3%)	6 (7.2%)
Family history VTE, *n* (%)	1 (14.3%)	7 (8.4%)
Unprovoked SVT, *n* (%)	2 (28.6%)	31 (37.4%)
Liver cirrhosis, *n* (%)	3 (42.9%)	11 (13.3%)
Solid cancer, *n* (%)	0 (0%)	6 (7.2%)
Myeloproliferative neoplasm, *n* (%)	1 (14.3%)	6 (7.2%)
Recent abdominal surgery, *n* (%)	0 (0%)	10 (12.1%)
Hormonal therapy, *n* (%)^*^	1 (33.3%)	10 (25.0%)
Inflammatory bowel disease, *n* (%)	0 (0%)	5 (6.0%)
JAK2 V617F mutation, *n* (%)^**^	1 (20.0%)	2 (5.4%)
Factor V Leiden or prothrombin G20210A mutation, *n* (%)^**^	1 (20.0%)	8 (16.0%)
Previous VKA treatment duration (months), median (IQR)	9.9 (3.2–12.9)	12.7 (7.2–24.0)
Time within therapeutic range (%), median (IQR)	68 (67–80)	62 (56–75)
Antiplatelet therapy during follow-up, *n* (%)	2 (28.6%)	12 (14.5%)

^*^Percentage calculated on the female population.

^**^Percentage calculated on the number of tested patients.

IQR = interquartile range, SVT = splanchnic vein thrombosis, VKA = vitamin K antagonist, and VTE = venous thromboembolism.

## Appendix

The following investigators and centers of the Italian Federation of Anticoagulation Clinics (FCSA) participated in this study (at least one patient enrolled):

- Daniela Poli, Thrombosis Centre, Department of Heart and Vessels, AOU Careggi, Firenze.
- Sophie Testa, Oriana Paoletti, Haemostasis and Thrombosis Centre, AO Istituti Ospitalieri, Cremona.
- Walter Ageno, Elena Rancan, Department of Clinical and Experimental Medicine, University of Insubria, Varese.
- Serena Rupoli, Irene Federici, and Lucia Canafoglia, Division of Hematology, Haemostasis and Thrombosis Center, AOU Ospedali Riuniti Umberto I, G.M. Lancisi, G. Salesi, Ancona, Italy.
- Rita Santoro, Hemophilia Center, Hemostasis and Thrombosis Unit, Azienda Ospedaliera “Pugliese-Ciaccio”, Catanzaro.
- Teresa Lerede, Anna Maggioni, and Anna Falanga, Department of Immunohematology and Transfusion Medicine, Hospital Papa Giovanni XXIII, Bergamo.
- Antonietta Piana, Department of Internal Medicine, Ospedale San Martino, Genoa.
- Monica Carpenedo, Department of Hematology and Transplant Unit, AO San Gerardo, Monza.
- Alberto Tosetto, Hemostasis and Thrombosis Center, Department of Hematology, S. Bortolo Hospital, Vicenza, Italy.
- Alberto Nicolini, Department of Internal Medicine, Arcispedale Santa Maria Nuova, Reggio Emilia.
- Piera Maria Ferrini, Dipartimento Emergenza-Urgenza e Area Medica e Specialistica, AOU Parma, Parma.
- Giuliana Martini, Spedali Civili di Brescia, Brescia.
- Catello Mangione, Ospedale di Galatina, Galatina.
- Laura Contino, Dipartimento Onco-Ematologico e Medicina Specialistica, Azienda Ospedaliera Nazionale SS. Antonio e Biagio e Cesare Arrigo, Alessandria.
- Carlo Bonfanti, Department of Transfusion Medicine and Haematology, Carlo Poma Hospital, Mantova.
- Paolo Gresele, Department of Internal Medicine, University of Perugia.
- Simona Pedrini, Morandini Rossella, Laboratory Service, Istituto Ospedaliero Fondazione Poliambulanza, Brescia.
- Lucia Marigo, Giovanni Nante, UOC Clinica Geriatrica, Università di Padova, Padova.
- Piera Sivera, A.O.O Ordine Mauriziano, Torino.
- Samantha Pasca, Center for Hemorrhagic and Thrombotic Diseases, University Hospital, Udine.
- Giuseppe Malcangi, Cosimo Pietro Ettorre, Centro Emofilia e Trombosi, Azienda Ospedaliero-Universitaria Policlinico, Bari.
- Cavallero Giobatta, Medicina Interna, Azienda Ospedaliera S. Croce e Carle, Cuneo.
- Pietro Falco, Thrombosis Centre, Hospice San Marco, Latina.
- Lucia Ruocco, Thrombosis Centre, Cisanello Hospital, Pisa.
- Carmelo Paparo, Ematologia e Coagulazione, Ospedale Maggiore, Chieri.
- Eugenio Bucherini, Angiology Unit, Civic Hospital, Faenza.
- Luciano Suriano, Rosanna Crisantemo, Servizio di Immunoematologia e Medicina Trasfusionale, Ospedale L. Bonomo, Andria.
- Luigi Ria, Department of Internal Medicine, Ospedale Sacro Cuore di Gesù, Gallipoli.
- Andrea Toma, UOC di Patologia Clinica, O.C. Arzignano, Arzignano.
- Antonio Insana, Department of Clinical Pathology, S. Croce Hospital, Moncalieri.
- Nello Zanatta, Divisione di Medicina Generale, Presidio Ospedaliero di Conegliano, Conegliano.
- Nicolò Zammataro, UO di Patologia Clinica, Ospedale Civile di Cuorgnè, Cuorgnè.
- Angela Maria Iannone, Servizio di Immunoematologia e Medicina Trasfusionale, Ospedale di Molfetta, Molfetta.
- Vincenzo Oriana, Centro Emofilia, Azienda Ospedaliera B.M. Morelli, Reggio Calabria.
- Mario Piergiulio Pezzo, Centro Trombosi, Ospedale di Suzzara, Suzzara.
- Giuseppe Isu. Thrombosis Centre, Department of Clinical Pathology, N.S. Bonaria Hospital, S. Gavino Monreale.
- Maurizio Molinatti, Centro Trombosi, Clinica Cellini, Torino.
